# Presentation of points of general discussion and voting among the speakers of the European Thyroid Association-Cancer Research Network (ETA-CRN) meeting in Lisbon, 2009, entitled ”European comments to ATA medullary thyroid cancer guidelines”

**DOI:** 10.1186/1756-6614-6-S1-S11

**Published:** 2013-03-14

**Authors:** Barbara Jarząb, Aleksandra Król, Kornelia Hasse-Lazar, Beata Jurecka-Lubieniecka

**Affiliations:** 1 Department of Nuclear Medicine and Endocrine Oncology, Maria Sklodowska-Curie Memorial Cancer Center and Institute of Oncology, Gliwice Branch, Wybrzeze Armii Krajowej 15, 44-101 Gliwice, Poland

## Abstract

The main subjects of discussion, held online within the ETA-CRN board invited 16 expert-panelists are shown. The ad hoc emerged ETA-CRN panel of experts (EPE) first congratulated Professor Kloos and the ATA Taskforce for the extensive work on medullary thyroid cancer, and appreciated discussing the ATA guidelines during the ETA-CRN meeting. As it was not possible for all experts to visit the meeting, they enclosed their comments in the online ETA forum. The overall intention was to evaluate certain discrepancies between the ATA guidelines and were biased European clinical practice.

All discussants were aware that the ATA guidelines had followed evidence based medicine rules; however, it was intended to reach an European consensus in this matter. The results of online voting among the EPE are shown.

We received answers from nine experts. The particular ATA guidelines devoted to the management of MTC ranged in agreement in 0/9 to 4/9. This did not reflect the general, good assessment of the guidelines, as of votes a set of questions.

The strongest discrepancies were found in assessment of the usefulness of pentagastrin (Peptavlon®) stimulated calcitonin secretion. The majority of the EPE (5/9) chose an option: “the increase of the basal Ct >100 ng/L means the substantial risk of MTC. However, there should also have been a recommendation for the grey zone 10-100 ng/L, where stimulation with pentagastrin is useful. The cut-off to perform stimulation test at ≤ 15-20 ng/L and values >100 ng/L means a significant suspicion of MTC”.

Similarly, attention from the EPE was raised towards the surgical procedures in MTC, particularly the extent and indications for lymph node surgical intervention. Four questions were related to the indications to lymphadenectomy and extent of surgery. The equal number (4/8) of EPE agreed with the ATA R61 and half of the ETA-CRN panel of experts disagreed because the indications to lymphadenectomy (Lx) depended in their opinion on the tumors detected by the Ct screening, in which prophylactic Lx might not be necessary.

“Notwithstanding the evidence based guidelines, their final acceptation requires unrestricted discussion and consideration of differences in clinical practice and experience between countries”.

## Introduction

The ATA (American Thyroid Association) MTC Guidelines published 2009 [1] raised a vivid discussion in Europe. This important paper constituted a stimulus for ETA (European Thyroid Association) members to arrange the ETA-CRN (ETA- Cancer Research Network) Meeting in Lisbon to discuss it.

The *ad hoc* emerged ETA-CRN panel of experts (EPE) first congratulated Professor R. Kloos and the ATA Taskforce for the extensive work on medullary thyroid cancer, reflected into the ATA guidelines and appreciated discussing these guidelines during the ETA-CRN meeting. As it was not possible for all experts to visit the meeting, the online ETA forum had been arranged and many ETA members enclosed their comments in the online ETA forum [2].

This paper summarizes the main subjects of discussion, shown in Additional files 1 and 2. The ETA-CRN board invited 16 expert-panelists to answer the questions which were formulated during the online discussion, on the basis of their experience and to comment on them during the ETA-CRN Meeting in Lisbon. All discussants were aware that the ATA guidelines had followed evidence based medicine rules; however, the online discussion was showed, because the guidelines required extensive changes of routine European clinical practice and it was intended to reach a European consensus in this matter. We received answers from nine experts.

As the reader may judge from Additional file 1, which was focused on management of MTC, the ETA-CRN panelists accepted the particular ATA guidelines devoted to the management from 0/9 to 4/9. This did not reflect the general, good assessment of the guidelines, as of votes a set of questions. The overall intention was to evaluate certain discrepancies between the ATA guidelines and were biased European clinical practice.

## Management of MTC

It was understood that the part of ATA Guidelines, described in Additional file 1, Table 1, considered rules of diagnosis and treatment of MTC, which were not different in management of sporadic and hereditary disease.

First, there was a discrepancy in the opinion on the reference range of basal calcitonin (Ct) level. The ATA Guideline R30 recommended that ”In the setting of an intact thyroid gland, Ct values should be interpreted in the setting of sex-specific reference ranges, at least in adults (Grade: B) “ [1], while the majority (6/9) of ETA-CRN panel of experts preferred to have one Ct reference range (Q1 option A), with normal Ct basal values ≤ 10 ng/l, despite being aware of sex-related differences.

Question 2 was related to ATA Guideline R52 which commented the usefulness of serum calcitonin estimation at the early diagnosis of MTC. This was an issue commented also in this Thyroid Research Supplement by Rosella Elisei and Cristina Romei [3]. The majority of European experts (5/9) agreed on the need for obligatory Ct estimation in nodular goiter and confirmed to perform that test. This was an expected discrepancy because the European consensus [4] on thyroid cancer recommended serum calcitonin measurement.

It seems, the strongest discrepancies were found in assessment of the usefulness of pentagastrin (Peptavlon®) stimulated calcitonin secretion. Thus, the ETA-CRN panel of experts did not agree on question 3, which was referring to Recommendation 52 (R52). “R52 defines a basal or stimulated serum Ct level > 100 ng/L, which should be interpreted as suspicious for MTC needing further evaluation if obtained”. This ATA guideline was supported in its full extent (option A) by one of the experts. The majority of the experts (5/9) chose option B: “Indeed, the increase of the basal Ct is >100 ng/L means the substantial risk of MTC. However, we should also have a recommendation for the grey zone 10-100 ng/L, and here the stimulation with pentagastrin is useful. The cut-off to perform stimulation test at ≤ 15-20 ng/L and values >100 ng/L means a significant suspicion of MTC. According to Scheuba et al. [5-9], the risk of MTC was 20% at a stimulated Ct>200 ng/L”. It is important to comment that these cut-offs were not valid for hereditary MTC but for sporadic cancer patients. The remaining 3/9 panelists agreed with the above statement but preferred to set the cut-off for stimulated Ct at 50 ng/L (Option C).

Similarly, the attention of the European experts were raised towards the surgical procedures in MTC recommended by ATA [1], particularly the extent and indications for lymph node surgical intervention. As seen from Additional file 1, questions Q4-Q8 were related to the indications to lymphadenectomy and extent of thyroid surgery (ATA Recommendations R61, 62, 66).

Among ETA-CRN panel of experts, the opinions were not unanimous. The equal number (4/8) agreed with the ATA R61 which stated that “Patients with known or highly suspected MTC with no evidence of advanced local invasion by the primary tumor, no evidence cervical lymph node metastases on physical examination and cervical US, and no evidence of distant metastases should undergo total thyroidectomy and prophylactic central compartment (level VI) neck dissection” and half of the ETA-CRN panel of experts disagreed because they stressed that the indications to lymphadenectomy (Lx) depended in their opinion on the tumors detected by the Ct screening, in which prophylactic Lx might not be necessary. Similar discrepancy within EPE was observed at the response to the Question 5, which was related to the ATA R59. Four from eight EPE agreed with the ATA Guideline and 4/8 chose the statement of Machens and Dralle [10] that liver Ct/ contrast enhanced MRI are necessary only when serum Ct> 1000 – 2000 pg/ml equal to 1000 – 2000 ng/L, because only then, the risk of distant dissemination is substantial.

The question Q6 (Additional file 1) referred to the indications to elective Lx in MTC. The ATA R62 states that if lymph node metastases are not detected by ultrasound, the elective (it means prophylactic) lateral Lx is not necessary. There was an important addition that a minority of the ATA Taskforce had favored prophylactic neck dissection when lymph node metastases were present in the adjacent paratracheal central compartment. There was also a minority (2/9) among the ETA-CRN panel of experts who expressed opinion (Q6 opinion A) “If no enlarged lymph node are detected, elective lateral lymph node dissection is not obligatory in MTC, irrespective of the status of the central neck lymph node”. However, the half of EPE (4/8) chose option B: “If no enlarged LN were detected, elective lateral lymph node dissection should be performed when lymph node metastases were present in the adjacent paratracheal central compartment” and the minority (2/8) of ETA-CRN panel of experts chose option C: “Elective lymph node dissection is obligatory in MTC, irrespective of the status of central neck lymph nodes”– one expert stressed it by the sentence “we perform bilateral neck dissection irrespective the lymph node status (skip lesion, micrometastases)”. Further, at question Q7, the ATA R66 stated that “in patients with extensive distant metastases a palliative neck operation might still be needed when there was pain, or evidence of tracheal compromise and the need to maintain a safe airway. They added also “Otherwise, in the setting of moderate to high volume extracervical disease, neck disease may be observed and surgery deferred. The ATA Task force opinion was not unanimous and the same was observed among ETA-CRN panel of experts: 2 of 9 of them chose option A, it means they had agreed with the deferral of local surgery in the setting of moderate to high volume extracervical MTC. The majority of ETA-CRN panel of experts (7/9) chose option B – they disagreed with the deferral of local surgery in the setting of moderate to high volume extracervical MTC.

The question Q8 (Additional file 1) referred to the indications to completion thyroidectomy (Tx) ATA R70-72. Two of eight European experts chose the option A: completion “is always indicated after unexpected diagnosis of MTC post less than total Tx, independently from MTC stage and should be completed by appropriate lymph node operation (at least central lymph node dissection, even if postoperative Ct is normal”. However, one of the expert surgeons added that completion Tx was always dependent on the basal/ stimulated Ct levels and genetic status and not on morphology. In a different spirit 4/8 experts chose option C: “Indications depend on the size of the primary tumors. The conditions listed in B may be valid only if solitary infracentimetric MTC was found”. And option B: “As proposed in ATA R70-71, completion thyroidectomy may be postponed after hemithyroidectomy, if unifocal intrathyroidal sporadic MTC was diagnosed, confined to the thyroid if no C-cell hyperplasia, negative surgical margin, and no suspicion for persistent disease on neck US and the basal serum Ct is below the upper normal of the reference range more than 2 months after surgery” was chosen by 2/8 experts.

The postoperative follow-up was discussed also in terms of usefulness of basal versus stimulated Ct estimation (Additional file 1, Question 9). Two of eight European experts were of the opinion that only basal Ct should be measured as the result of postoperative pentagastrin test does not usually modify the follow-up strategy when basal calcitonin is low. 4/9 chose option B, recommending rather stimulated Ct as more sensitive and 5/9 chose option C (one expert chose simultaneously option B and C). The option C was a compromise between radical options A and B and recommended the pentagastrin test at least at first postoperative evaluation if basal Ct was low to confirm the full success of the operation. It was explained that patients with normal basal, but increased stimulated Ct did not require additional treatment however, they might not be regarded as completely free of disease and required more cautious monitoring. Finally, in the postoperative follow-up, the high sensitivity of Ct testing was related to the difficulties in the localization of the persistent or recurrent disease, especially if Ct level was only moderately increased.

In question Q10 the European experts evaluated the ATA R75, which proposed the cut-off < 150 pg/ml (equal to <150 ng/L), below which the postoperative imaging might be limited to sonography only. Three of nine experts had agreed with the ATA recommendation R75 (Q10, option A). Six of nine ones preferred R76, which stated that post-operative MTC patients with detectable serum Ct levels < 150 ng/L might be considered for additional imaging with Ct or MRI to serve as baseline examinations for future comparison even though these studies were usually negative.

## Hereditary MTC

The other part of online discussion was related to the ATA Guidelines that were devoted to the issues of hereditary MTC, meaning indications to RET genetic diagnostics and treatment of RET mutations carriers.

The EPE consultations were reflected in Additional file 2. The indications for RET testing in patients with primary C cell hyperplasia, contained in the ATA R1, was accepted by 5/9 European experts Additional file 2; Question 20, option A) while the other 4/9 protested, because the risk of finding RET mutations in a C cell hyperplasia patient was assumed to be low.

Also, there has been a discussion on the early symptoms of MEN 2B (Question 21). Three of nine experts accepted the ATA R2, which recommended RET testing in cases of intestinal gangliomatosis (option A), while the majority (6/9) chose option C: “Both bumpy lips with mucosal neuromas and corneal fibres should be also considered. Thickening of corneal fibres is very frequent, with a prevalence of 69% in MEN 2B [11] and 29% in MEN 2A [12]”. Tearless crying of small children was not accepted as an indication for RET testing without any other MEN 2 symptoms by 7/9 experts because they needed more evidence (Question 22, option C). However, they found this sign worth mentioning in the European comments to ATA MTC Guidelines.

The ATA R4 recommended RET testing in cases of lichen planus and it was accepted by 5/9 EPE (Question 23, Option A). In fact, further 2/9 experts did not intend to propose a change in ATA R4 recommendation (option C). The R6-R8 Guidelines recommended the ATA risk classes and this new subdivision was accepted by nearly all experts (8/9), but 7 of 9 preferred option B and stressed the necessity to comment it (“To include Ct levels into decision making seems mandatory”). One of the experts argued for a statement to discourage delayed thyroid surgery in RET mutation carriers and this statement was supported by a letter by the Dutch experts [2] (please visit: http://eta-crn.eurothyroid.com for the further reference).

This discussion, encompassed by questions 25-28 has been reflected in a separate article in this Thyroid Research Supplement [13]. The problem of indications to RET testing has been widened by the question of Hirschprung disease (see question 30, Additional file 2). The ATA R10 recommended to consider RET testing in all patients with Hirschprung disease and it was supported by 2 of 9 EPE (Additional file 2, Question 30, option A.). However, the majority of ETA-CRN panel of experts (5/9) chose option B: “Hirschsprung disease (HD) is very common (about 1/5000 births) and at least 10 related genes have been identified. Activating RET mutations have been found in exons 10 and 11 only in in about 2% of cases [14] and this prevalence is too low to recommend the testing as grade A recommendation”.

Questions Q31-Q32 were devoted to the problem of extent of DNA testing. The ATA R11 recommended it either a single or multicentered approach and this was supported by 1 of 9 ETA-CRN panel of experts. The majority (5/9) chose option B: “Systematic screening for RET mutations in exon 10, 11, 13, 14, 15, and 16 represents the current gold standard and should be completed by exon 8 analysis in all regions where it was described to be present” while 2/9 meant testing of RET exon 8 as unnecessary in routine DNA diagnosis, as in fact, ATA R11 proposed.

It was also the intention of ETA-CRN panel of experts to widen the discussion by the problem of RET polymorphisms (Additional file 2, Question 33) which was missed by ATA Guidelines. However, the majority of experts 7/9 were of opinion that the data on RET polymorphism are still insufficient to draw any conclusion (option B) and supported the ATA Task force view.

The question of surgical management of RET carriers is reflected in Additional file 2 (Questions Q33, Q35, Q36). The conclusions of the discussion may be summarized in the “ both DNA and calcitonin level” concept [10].

## Conclusion

These data serve to illustrate the European consensus on acceptation of ATA MTC Guidelines. The conclusion from the “Result and summary of voting among the audience during presentation and discussion of Medullary Thyroid Carcinoma Clinical Guidelines prepared by American Thyroid Association” [15] should be cited:

“European expert opinion leaders and an audience of specialist in treatment of Medullary Carcinoma welcomes the American Guidelines on the management of MTC, but simultaneously only partially agrees with some of the expert statements. The results of the survey prior to the meeting were biased in that the presenters were selected for presenting the results, but the audience was present upon open invitation through scientific channels. Notwithstanding the evidence based guidelines, their final acceptation requires unrestricted discussion and consideration of differences in clinical practice and experience between countries. These discussion and results subsequently formed the basis for establishing a task force within the ETA, and consequent publication of European guideline mainly for the treatment aspects of metastatic MTC.”

## Competing interests

No competing interests exist for the authors.

## Supplementary Material

Additional file 1 Table 1. Diagnosis and management of MTC – questions to experts and their answers Click here for file

Additional file 2 Table 2. Hereditary MTC – questions to experts and their answers Click here for file

## Declarations

This article has been published as part of *Thyroid Research* Volume 6 Supplement 1, 2013: European comments on Medullary Thyroid Cancer Management Guidelines of the American Thyroid Association. The full contents of the supplement are available online at http://www.thyroidresearchjournal.com/supplements/6/S1. Publication of this supplement has been supported by the European Thyroid Association-Cancer Research Network.
