# Supplementary material for: Presentation of points of general discussion and voting among the speakers of the European Thyroid Association-Cancer Research Network (ETA-CRN) meeting in Lisbon, 2009, entitled ”European comments to ATA medullary thyroid cancer guidelines”
Source: Thyroid Res. 2013 Mar 14;6(Suppl 1):S11. doi: 10.1186/1756-6614-6-S1-S11 (PMC3599728; doi:10.1186/1756-6614-6-S1-S11)
Supplement: Additional file 1 — Table 1. Diagnosis and management of MTC – questions to experts and their answers [file 1756-6614-6-S1-S11-S1.doc]

| **1.**  **Table 1. Diagnosis and management of MTC - questions to experts**   - *comments added by the experts are shown in italics*     **What is your opinion on the reference range of basal Ct?** | **1** | **2** | **3** | **4** | **5** | **6** | **7** | **8** | **9** | **10** | **11** | **12** | **13** | **14** | **15** | **16** |
| --- | --- | --- | --- | --- | --- | --- | --- | --- | --- | --- | --- | --- | --- | --- | --- | --- |
| A. Although there are some sex-related differences, with higher values seen in men, I prefer to have one reference range, with normal basal values ≤ 10 ng/L | **x** |  |  |  |  | **x** | **x** | **x** |  |  | **x** |  | **x** |  |  |  |
| B. It is necessary to interpret basal Ct separately in women and in men |  |  |  |  |  |  |  |  | **x** |  |  |  | **x** | **x** |  | **x** |
| **2.**  **The ATA Guideline R52 defers the recommended approach to thyroid nodules, including fine needle aspiration biopsy and serum Ct testing, to the ATA Guideline that addresses thyroid nodules. Given the fact that the European consensus for the management of patients with differentiated thyroid carcinoma of the follicular epithelium recommends Ct testing in nodular disease, and that this consensus has also been endorsed by the ETA, in ETA comments the referral will be made to this European consensus** | **1** | **2** | **3** | **4** | **5** | **6** | **7** | **8** | **9** | **10** | **11** | **12** | **13** | **14** | **15** | **16** |
| A. I accept the ATA guideline in its full extent |  |  |  |  |  |  |  |  |  |  |  |  |  |  |  |  |
| B. I agree with the obligatory Ct estimation in all patients with thyroid nodules and perform it | **x** |  |  |  |  | **x** |  |  |  |  | **x** |  | **x** | **x** |  |  |
| C. I agree the obligatory Ct estimation in all patients with thyroid nodules but due to the financial limitations I am unable to perform it in all my patients |  |  |  |  |  |  |  |  |  |  |  |  |  |  |  |  |
| D. Considering the low prevalence of MCT I think that obligatory Ct estimation needs still better evaluation of its benefits, not only health related but also cost-related |  |  |  |  |  |  | **x** | **x** | **x** |  |  |  |  |  |  | **x** |
| **3.**  **R52 defines a basal or stimulated* serum Ct level > 100 ng/L, which should be interpreted as suspicious for MTC and further evaluation if obtained. Your opinion is:** | **1** | **2** | **3** | **4** | **5** | **6** | **7** | **8** | **9** | **10** | **11** | **12** | **13** | **14** | **15** | **16** |
| A. I accept the ATA guideline in its full extent |  |  |  |  |  |  |  |  |  |  |  |  |  | **x** |  |  |
| B. Indeed, the increase of the basal Ct is >100 ng/L means the substantial risk of MTC. However, we should have also recommendation for the grey zone 10-100 ng/L and here the stimulation with pentagastrin is useful. The cut-off to perform stimulation test at ≤ 15-20 ng/L and values >100 ng/L mean significant suspicion of MTC. According to [9], risk of MTC is 20% at the stimulated Ct>200 ng/L | **x** |  |  |  |  |  |  | **x** | **x** |  |  |  | **x** |  |  | **x** |
| C. I agree with B but prefer to set the cut-off for stimulated Ct at 50 ng/L |  |  |  |  |  | **x** | **x** |  |  |  | **x** |  |  |  |  |  |
| D. I agree with B but prefer to set the cut-off for stimulated Ct at 200 ng/L or even greater |  |  |  |  |  |  |  |  |  |  |  |  |  |  |  |  |
| **4.**  **The ATA R61 states “Patients with known or highly suspected MTC with no evidence of advanced local invasion by the primary tumor, no evidence cervical lymph node metastases on physical examination and cervical US, and no evidence of distant metastases should undergo total thyroidectomy and prophylactic central compartment (level VI) neck dissection” (please note that this recommendation does not consider diagnosed RET carriers referred for prophylactic thyroidectomy)** | **1** | **2** | **3** | **4** | **5** | **6** | **7** | **8** | **9** | **10** | **11** | **12** | **13** | **14** | **15** | **16** |
| A. I agree because the absence of any enlarged lymph node by ultrasound does not exclude the presence of lymph node metastases in MTC. A central neck dissection in the case of elevated calcitonin levels is always necessary, even in preclinical disease. |  |  |  |  |  | **x** |  |  |  |  | **x** |  | **x** | **x** |  |  |
| B. I disagree because it depends on the tumor stage. In very small tumors detected by calcitonin screening prophylactic central lymph node dissection may not be necessary. | **x** |  |  |  |  |  | **x** | **x** | **x** |  |  |  |  |  |  |  |
| **5.**  **Preoperative chest CT, neck CT, and 3 phase contrast enhanced multidetector liver CT or contrast enhanced MRI is recommended for all patients with suspected MTC when local lymph node metastases are detected (N1), or the serum Ct is > 400 pg/ml** | **1** | **2** | **3** | **4** | **5** | **6** | **7** | **8** | **9** | **10** | **11** | **12** | **13** | **14** | **15** | **16** |
| A. I agree with the ATA guideline |  |  |  |  |  |  |  |  | **x** |  | **x** |  |  | **x** |  | **x** |
| B.  I think that preoperative chest neck CT and 3 phase liver CT/contrast enhanced MRI are necessary only when serum Ct is larger than 1000-2000 pg/ml, because only then, according to Machens and Dralle (Thyroid 2009[10]), the risk of distant dissemination is substantial | **x** |  |  |  |  | **x** | **x** | **x** |  |  |  |  |  |  |  |  |
| C. These examinations are obligatory in each case excluding prophylactic thyroidectomy |  |  |  |  |  |  |  |  |  |  |  |  |  |  |  |  |
| **6.**  **R62 states that if lymph node metastases are not detected by ultrasound, the elective lateral lymphadenectomy is not necessary. However:**  **“A minority of the Task Force favored prophylactic lateral neck dissection when lymph node metastases were present in the adjacent paratracheal central compartment”.**  **Your opinion is:** | **1** | **2** | **3** | **4** | **5** | **6** | **7** | **8** | **9** | **10** | **11** | **12** | **13** | **14** | **15** | **16** |
| A. If no enlarged LN are detected, elective lateral lymph node dissection is not obligatory in MTC, irrespective of the status of central neck lymph node |  |  |  |  |  |  |  |  |  |  | **x** |  |  | **x** |  |  |
| B. If no enlarged LN were detected, elective lateral lymph node dissection should be performed when lymph node metastases were present in the adjacent paratracheal central compartment | **x** |  |  |  |  | **x** | **x** | **x** |  |  |  |  |  |  |  |  |
| C. Elective lymph node dissection is obligatory in MTC, irrespective of the status of central neck lymph nodes - *we perform bilateral lateral neck dissection irrespective the lymph node status (skip lesion, micrometastases)** |  |  |  |  |  |  |  |  | **x** |  |  |  | **x** |  |  |  |
| D. I have no own opinion in this matter |  |  |  |  |  |  |  |  |  |  |  |  |  |  |  |  |
| **7.**  **R66 states that in patients with extensive distant metastases a palliative neck operation may still be needed when there is pain, or evidence of tracheal compromise and the need to maintain a safe airway. Otherwise, in the setting of moderate to high volume extra-cervical disease, neck disease may be observed and surgery deferred (Task Force opinion was not unanimous).** | **1** | **2** | **3** | **4** | **5** | **6** | **7** | **8** | **9** | **10** | **11** | **12** | **13** | **14** | **15** | **16** |
| A. I agree with the deferral of local surgery in the setting of moderate to high volume extra-cervical MTC |  |  |  |  |  |  |  |  | **x** |  |  |  |  | **x** |  |  |
| B. I disagree with the deferral of local surgery in this setting. If doable, a palliative neck operation may lower pain, tracheal compromise and helps to maintain a safe airway. | **x** |  |  |  |  | **x** | **x** | **x** |  |  | **x** |  | **x** |  |  | **x** |
| C. I have no personal opinion in this matter |  |  |  |  |  |  |  |  |  |  |  |  |  |  |  |  |
| **8.**  **Completion thyroidectomy -** *always dependent on the b/S CT levels and genetic status NOT on morphology** | **1** | **2** | **3** | **4** | **5** | **6** | **7** | **8** | **9** | **10** | **11** | **12** | **13** | **14** | **15** | **16** |
| A. Is always indicated after unexpected diagnosis of MTC post less than total thyroidectomy, independently from MTC stage and should be completed by the appropriate lymph node operation (at least central LND, even if postoperative Ct is normal |  |  |  |  |  | **x** |  |  |  |  | **x** |  |  |  |  |  |
| B. As proposed in ATA R70-71, completion thyroidectomy may be postponed after hemithyroidectomy, if unifocal intrathyroidal sporadic MTC was diagnosed, confined to the thyroid if no C-cell hyperplasia, negative surgical margin, and no suspicion for persistent disease on neck US and the basal serum Ct is below the upper normal of the reference range more than 2 months after surgery |  |  |  |  |  |  | **x** |  |  |  |  |  |  | **x** |  |  |
| C. Indications depend on the size of the primary tumors. The conditions listed in B may be valid only if solitary infracentimetric MTC was found | **x** |  |  |  |  |  |  | **x** | **x** |  |  |  |  |  |  | **x** |
| D. I have no own opinion in this matter |  |  |  |  |  |  |  |  |  |  |  |  |  |  |  |  |
| **9.**  **Postoperative follow-up should base on Ct and CEA estimation (R73-74)** | **1** | **2** | **3** | **4** | **5** | **6** | **7** | **8** | **9** | **10** | **11** | **12** | **13** | **14** | **15** | **16** |
| A.  Only basal Ct should be measured as the result of postoperative pentagastrin test does not usually modify the follow-up strategy when basal calcitonin is low. | **x** |  |  |  |  |  |  |  | **x** |  |  |  |  |  |  |  |
| B.   Stimulated Ct is more sensitive than basal level and should be regularly performed postoperatively (in yearly intervals) |  |  |  |  |  | **x** |  |  |  |  |  |  | **x** |  |  | **x** |
| C.   Pentagastrin test should be performed at least at first postoperative evaluation if basal Ct is low to confirm the full success of the operation. Patients with normal basal but increased stimulated Ct do not require additional treatment in this moment, however, they may not be regarded as completely free of the disease and require more cautious monitoring. |  |  |  |  |  |  | **x** | **x** |  |  | **x** |  |  | **x** |  | **x** |
| D. I have no own opinion in this matter |  |  |  |  |  |  |  |  |  |  |  |  |  |  |  |  |
| **10.**  **In the postoperative follow-up the high sensitivity of Ct testing implies that the localization of the persistent recurrent disease may be impossible when Ct level is only moderately increased. The ATA R75 guideline proposes the cut-of of <150 pg/ml, below which postoperative imaging may be limited to US only. Do you agree?** | **1** | **2** | **3** | **4** | **5** | **6** | **7** | **8** | **9** | **10** | **11** | **12** | **13** | **14** | **15** | **16** |
| A.  I agree with R75. Additional imaging can be deferred and subsequently implemented should the serum Ct rise over time. |  |  |  |  |  |  | **x** | **x** |  |  |  |  | **x** |  |  |  |
| B. I prefer R76 which states that post-operative MTC patients with detectable serum Ct levels that are <150 pg/ml may be considered for additional imaging (CT/MRI in addition to US) to serve as baseline examinations for future comparison even though these studies are usually negative | **x** |  |  |  |  | **x** |  |  | **x** |  | **x** |  |  | **x** |  | **x** |
| C. I think that postoperative imaging is necessary in every case post surgery, even in patients with undetectable CT, to serve as baseline examinations |  |  |  |  |  |  |  |  |  |  |  |  |  |  |  |  |
| D. I have no own opinion in this matter |  |  |  |  |  |  |  |  |  |  |  |  |  |  |  |  |
| **11.**  **R78 states that in the absence of residual anatomically identifiable disease (neck US and CT) in a thyroidectomized patient with a measurable Ct level who has not previously undergone a level VI compartmental dissection, an empiric central compartment dissection may be considered but remains controversial. Your opinion:** | **1** | **2** | **3** | **4** | **5** | **6** | **7** | **8** | **9** | **10** | **11** | **12** | **13** | **14** | **15** | **16** |
| A. I agree with the ATA guideline that an empiric central LND may not be successful if the MTC focus is known only by the increased Ct and has not been localized |  |  |  |  |  |  |  |  |  |  | **x** |  |  | **x** |  |  |
| B. If central LND has not been performed previously, it should be performed, as the probability of lymph node metastases in this compartment is high despite the lack of enlarged lymph nodes visible on US. | **x** |  |  |  |  | **x** | **x** | **x** | **x** |  |  |  | **x** |  |  | **x** |
| C. I have no own opinion in this matter |  |  |  |  |  |  |  |  |  |  |  |  |  |  |  |  |
| **12.**  **Postoperative adjuvant EBRT to the neck and mediastinum may be considered in patients who are found to have microscopic positive margin(s) (R1 resection) following surgery for moderate to high volume disease involving the central compartment (level VI) and one or both lateral neck compartments (levels 2A-V)** | **1** | **2** | **3** | **4** | **5** | **6** | **7** | **8** | **9** | **10** | **11** | **12** | **13** | **14** | **15** | **16** |
| A. I agree with the ATA guideline |  |  |  |  |  |  |  |  |  |  |  |  |  |  |  |  |
| B. This recommendation may be accepted only in patients with evidence of incomplete resection (R2 resection) |  |  |  |  | **No answers given** | | | | | | | | |  |  |  |
| C. I do not agree, as EBRT will lead to considerable toxicity without any evidence for improved overall survival |  |  |  |  |  |  |  |  |  |  |  |  |  |  |  |  |
| **13.**  **The routine use of cytotoxic chemotherapy should be discouraged in patients with MTC. It may be considered for selected patients with rapidly progressive disease not amenable to clinical trials** | **1** | **2** | **3** | **4** | **5** | **6** | **7** | **8** | **9** | **10** | **11** | **12** | **13** | **14** | **15** | **16** |
| A. I agree with the ATA guideline |  |  |  |  |  |  |  |  |  |  |  |  |  |  |  |  |
| B. I do not agree, as cytotoxic chemotherapy is standard of care in patients with metastatic MTC |  |  |  |  |  |  | **No answers given** | | | | | |  |  |  |  |
| **12.**  **R81 states that In post-operative MTC patients with serum Ct levels >150 pg/ml with small (<1 cm) locoregional metastatic disease that is asymptomatic and non-threatening, and distant metastases are present, immediate intervention towards the locoregional disease is of unknown benefit and such lymph nodes may be observed. Do you agree with this guideline?** | **1** | **2** | **3** | **4** | **5** | **6** | **7** | **8** | **9** | **10** | **11** | **12** | **13** | **14** | **15** | **16** |
| A. I agree with the ATA guideline |  |  |  |  |  | **x** |  |  |  |  | **x** |  |  | **x** |  |  |
| B. This recommendation may be accepted only if central and lateral lymph node dissection was previously done | **x** |  |  |  |  |  | **x** | **x** | **x** |  |  |  | **x** |  |  | **x** |
| C. I do not agree, it is always worth to remove the known locoregional tumor, even if distant MTC metastases are present, provided they do not constitute the immediate life threatening |  |  |  |  |  |  |  |  |  |  |  |  |  |  |  |  |
| **13.**  **The role of FDG PET imaging in primary evaluation of MCT prior to surgery may be defined by:** | **1** | **2** | **3** | **4** | **5** | **6** | **7** | **8** | **9** | **10** | **11** | **12** | **13** | **14** | **15** | **16** |
| A. I agree with ATA guidelines that FDG PET is not recommended in primary preoperative evaluation | **x** |  |  |  |  | **x** | **x** | **x** | **x** |  |  |  | **x** | **x** |  | **x** |
| B. I do not agree with ATA guidelines because FDG PET helps in preoperative staging |  |  |  |  |  |  |  |  |  |  |  |  |  |  |  |  |
| C. Receptor PET imaging is more useful in primary MCT staging and should be recommended |  |  |  |  |  |  |  |  |  |  |  |  |  |  |  |  |
| D. I do not have own opinion in this matter |  |  |  |  |  |  |  |  |  |  | **x** |  |  |  |  |  |
| **14.**  **The role of FDG PET imaging in postoperative evaluation of MCT prior to surgery may be defined by:** | **1** | **2** | **3** | **4** | **5** | **6** | **7** | **8** | **9** | **10** | **11** | **12** | **13** | **14** | **15** | **16** |
| A. FDG PET should be performed in cases of asymptomatic hypercalcitoninaemia to localize foci of MTC |  |  |  |  |  |  |  |  |  |  |  |  |  |  |  |  |
| B. FDG PET is not sufficiently sensitive for detection of small metastatic foci | **x** |  |  |  |  | **x** |  |  | **x** |  |  |  |  | **x** |  |  |
| C. FDG PET should be apply to detect metastatic foci only if Ct>400 ng/L |  |  |  |  |  |  | **x** | **x** |  |  |  |  | **x** |  |  | **x** |
| D. I do not have own opinion in this matter |  |  |  |  |  |  |  |  |  |  | **x** |  |  |  |  |  |
| **15.**  **MIBG therapy and peptide receptor radiotherapy** | **1** | **2** | **3** | **4** | **5** | **6** | **7** | **8** | **9** | **10** | **11** | **12** | **13** | **14** | **15** | **16** |
| A. May be useful in palliative therapy of advanced MCT |  |  |  |  |  | **x** | **x** | **x** |  |  | **x** |  |  |  |  | **x** |
| B. Do not play any role in therapy of advanced MCT | **x** |  |  |  |  |  |  |  | **x** |  |  |  |  | **x** |  |  |
| C. I have no own opinion in this matter |  |  |  |  |  |  |  |  |  |  |  |  | **x** |  |  |  |
| **16.**  **Somatostatin analogues are not recommended by ATA guidelines as antitumor agents in MTC** | **1** | **2** | **3** | **4** | **5** | **6** | **7** | **8** | **9** | **10** | **11** | **12** | **13** | **14** | **15** | **16** |
| A. I agree with ATA guideline as the published data do not document anti-tumor activity in MCT | **x** |  |  |  |  | **x** |  |  | **x** |  |  |  |  | **x** |  | **x** |
| B. I agree with the lack of antitumoral effect but there is also another indication in MCT associated with symptomatic diarrhea or Cushing syndrome where somatostatin analogues are worth to be evaluated in patients with documenter SS receptor expression |  |  |  |  |  |  | **x** | **x** |  |  | **x** |  | **x** |  |  | **x** |
| C. I have no own opinion in this matter |  |  |  |  |  |  |  |  |  |  |  |  |  |  |  |  |
| **17.**  **Asymptomatic residual hypercalcitoninaemia and pregnancy** | **1** | **2** | **3** | **4** | **5** | **6** | **7** | **8** | **9** | **10** | **11** | **12** | **13** | **14** | **15** | **16** |
| A. Pregnancy should be contraindicated in any case of persistent MTC, also when the increase of Ct level is not concomitted by positive disease imaging |  |  |  |  |  |  |  |  |  |  |  |  | **x** |  |  |  |
| B. Pregnancy may be considered in persistent not progressing hypercalcitoninemia if doubling time of Ct is more than 2 years |  |  |  |  |  | **x** | **x** |  |  |  |  |  |  |  |  | **x** |
| C. Increase of Ct level without localization of the disease should not constitute an absolute contraindication to pregnancy | **x** |  |  |  |  |  |  |  | **x** |  | **x** |  |  | **x** |  | **x** |
