# Supplementary material for: Presentation of points of general discussion and voting among the speakers of the European Thyroid Association-Cancer Research Network (ETA-CRN) meeting in Lisbon, 2009, entitled ”European comments to ATA medullary thyroid cancer guidelines”
Source: Thyroid Res. 2013 Mar 14;6(Suppl 1):S11. doi: 10.1186/1756-6614-6-S1-S11 (PMC3599728; doi:10.1186/1756-6614-6-S1-S11)
Supplement: Additional file 2 — Table 2. Hereditary MTC – questions to experts and their answers [file 1756-6614-6-S1-S11-S2.doc]

| **Table 2. Hereditary MTC - questions to experts** |  |  |  |  |  |  |  |  |  |  |  |  |  |  |  |  |
| --- | --- | --- | --- | --- | --- | --- | --- | --- | --- | --- | --- | --- | --- | --- | --- | --- |
| **20.**  **Ad R1. Do you accept the indication for RET testing in patients with personal medical history of primary C hyperplasia in whom no diagnosis of MTC has been made?** | **1** | **2** | **3** | **4** | **5** | **6** | **7** | **8** | **9** | **10** | **11** | **12** | **13** | **14** | **15** | **16** |
| A. Yes, because new carriers of RET germline mutation can be detected in this way (concordant with ATA MTC Guidelines) |  |  |  |  |  | **x** |  |  | **x** |  |  |  | **x** | **x** |  | **x** |
| B. No, because the risk of finding a germline RET mutation carrier is too low (lower than 5%, according to Scheuba et al., 2009 [7,9]) | **x** |  |  |  |  |  | **x** | **x** |  |  | **x** |  |  |  |  |  |
| C. No, because the benefit is unproven |  |  |  |  |  |  |  |  |  |  |  |  |  |  |  |  |
| **21.**  **Ad R2 Considering the fact that up to 90% of MEN2 B cases are caused by *de novo* mutations, (Brauckhoff et al. [2]), do you consider the indications listed in ATA R2 sufficient ? (ATA recommends RET testing in cases of intestinal ganglioneuromatosis only)** | **1** | **2** | **3** | **4** | **5** | **6** | **7** | **8** | **9** | **10** | **11** | **12** | **13** | **14** | **15** | **16** |
| A. Yes, because intestinal ganglioneuromatosis is often diagnosed before the diagnosis of MEN2B (concordant with ATA MTC Guidelines) |  |  |  |  |  |  |  |  |  |  | **x** |  | **x** | **x** |  |  |
| B. No, because bumpy lips with mucosal neuromas should also be considered as indication | **x** |  |  |  |  |  |  |  |  |  |  |  |  |  |  |  |
| C. Both bumpy lips with mucosal neuromas and corneal fibres should be also considered. Thickening of corneal fibres is very frequent, with a prevalence of 69% in MEN 2B [11] and 29% in MEN 2A [12] |  |  |  |  |  | **x** | **x** | **x** | **x** |  |  |  |  |  |  | **x** |
| **22.**  **Ad R2 Tearless crying is observed in up to 85% children with MEN2B (Brauckhoff and Gimm [16]). Do you accept inclusion of this sign as indication for RET testing in small children without any other MEN2 symptoms and with negative family history?** | **1** | **2** | **3** | **4** | **5** | **6** | **7** | **8** | **9** | **10** | **11** | **12** | **13** | **14** | **15** | **16** |
| A. Yes, because it is necessary to diagnose de novo MEN2 cases as soon as possible |  |  |  |  |  |  |  |  | **x** |  |  |  |  | **x** |  |  |
| B. No, because the prevalence of the tearless crying in the normal population is not well defined and the risk of false positivity is substantial |  |  |  |  |  |  |  |  |  |  |  |  |  |  |  | **x** |
| C. No, because more data is necessary. However, I find this sign promising and worth mentioning in the comments. | **x** |  |  |  |  | **x** | **x** | **x** |  |  | **x** |  | **x** |  |  | **x** |
| **23.**  **Ad R4 - The ATA R4 recommends RET testing in cases of lichen planus as Grade B recommendation. The proposal is to strengthen this recommendation into the following statement: Lichen planus amyloidosis or pruritus in the central upper back may indicate the presence of a RET mutation and should prompt CT measurement in adults and genetic testing in children (possibly after 4-mm punch biopsy and staining with thioflavin T in order to identify amyloid deposits). Do you accept this proposal?** | **1** | **2** | **3** | **4** | **5** | **6** | **7** | **8** | **9** | **10** | **11** | **12** | **13** | **14** | **15** | **16** |
| A.  Yes. Indeed lichen planus in MEN has been reported in several papers from the literature and is more frequent than suspected, up to 36% of MEN2A cases (Verga et al., 2003 [17]). Moreover, lichen planus is associated not only to codon 634, but also to codon 804 (Rothberg et al., 2009 [18]) |  |  |  |  |  | **x** |  |  | **x** |  | **x** |  | **x** | **x** |  |  |
| B. No, I prefer the more cautious standpoint of ATA Guidelines |  |  |  |  |  |  |  |  |  |  |  |  |  |  |  | **x** |
| C. I propose to mention it in the comments but without changing the ATA R4 recommendation. | **x** |  |  |  |  |  |  | **x** |  |  |  |  |  |  |  |  |
| **24.**  **Ad Table 6 - ATA gives in Table 6 a division of RET germline carriers into 4 risk categories (ATA A – the lowest risk, ATA D: the highest risk) which differs from the previous division into 3 risk categories (Brandi et al. 2001 [19]) by separation of the high risk related to RET 634 and RET 630 (ATA B class) and the moderately high risk of other exon 10 mutations (ATA A class). What is your opinion on this modification?** | **1** | **2** | **3** | **4** | **5** | **6** | **7** | **8** | **9** | **10** | **11** | **12** | **13** | **14** | **15** | **16** |
| A. I accept it fully, it rationalizes the differences in phenotype and is clinically relevant | **x** |  |  |  |  | **x** | **x** | **x** | **x** |  | **x** |  |  | **x** |  | **x** |
| B. I do not accept it and prefer the previous division of risk into three categories , as presented by Brandi et al 2001 [19] |  |  |  |  |  |  |  |  |  |  |  |  |  |  |  |  |
| C. I accept it but would prefer a 5 categories division, with additional category of the lowest risk of the RET mutations which haven’t been [proven fully (for example RET 649) |  |  |  |  |  |  |  |  |  |  |  |  |  |  |  |  |
| **25.**  **Ad R6-8: ATA guidelines recommend to consider ATA risk class and age and allow to “delay prophylactic thyroidectomy beyond age 5 years in patients with ATA Level A and B *RET* mutations in the setting of a normal annual basal +/- stimulated* serum Ct, normal annual neck US, less aggressive MTC family history and family preference. Surgery is indicated if all of these features are not present”. Simultaneously the state “For higher risk mutations (ATA Level B), consider treatment before age 5 years in an experienced tertiary care setting, regardless of other factors”. My general opinion on timing of prophylactic thyroidectomy in RET mutation carriers is:** | **1** | **2** | **3** | **4** | **5** | **6** | **7** | **8** | **9** | **10** | **11** | **12** | **13** | **14** | **15** | **16** |
| A. I accept this statement fully |  |  |  |  |  |  |  |  |  |  |  |  |  | **x** |  |  |
| B. I prefer to comment as expressed by Machens and Dralle [10]: Age as major criterion for timing of prophylactic thyroidectomy is difficult to assess due to the wide spectrum of C-cell transformation from CCH to MTC even within defined risk groups, and even with families. The reason for that is that clinical manifestation of C-cell-disease does not depend only on RET mutation but also on second hits and some ill-defined modifying factors. To include calcitonin levels into decision making seems mandatory. | **x** |  |  |  |  | **x** |  | **x** | **x** |  | **x** |  | **x** |  |  | **x** |
| C. I argue for a statement to discourage delayed thyroid surgery and subsequent annual (basal and/or stimulated) calcitonin testing and ultrasound as expressed by the Dutch MTC Group: Refraining from thyroid surgery before the age of 5 years in ATA-A-D RET mutation carriers is not an option in patients with elevated basal or stimulated calcitonin levels. Although timing of prophylactic surgery based on genotype only is far from optimal because evidently some patients will be overtreated, we think that prophylactic thyroidectomy should be recommended before the youngest age reported in the literature, according to the genotype. Delaying prophylactic thyroidectomy beyond 3-5 years of life may be only justified because of family preference [2]. |  |  |  |  |  |  | **x** |  |  |  |  |  |  |  |  |  |
| **26.**  **Ad R6-8 - If you selected 23B and argued for evaluation of Ct , please indicate what is your opinion for use of stimulated Ct for decision on timing of prophylactic thyroidectomy** | **1** | **2** | **3** | **4** | **5** | **6** | **7** | **8** | **9** | **10** | **11** | **12** | **13** | **14** | **15** | **16** |
| A. I agree with the statement in ATA guidelines. Most experts believe that there is rarely a need for stimulated CT testing. Using repetitive pentagastrin - that is available in Europe - stimulation in patients with a ATA level A or B RET mutation is also a burden for a young patient, with a risk of not being cured after surgery. |  |  |  |  |  |  |  |  | **x** |  |  |  |  | **x** |  |  |
| B. Moreover, in addition to A, sensitive calcitonin assays have to be proven their value in these circumstances |  |  |  |  |  |  |  |  |  |  |  |  |  |  |  |  |
| C. The value of the stimulated Ct levels in this setting is to determine the dynamics of C-cell transformation in order to avoid underestimation but also overestimation of this process, i.e. to avoid both undertreatment and overtreatment in regard to the time and extent of operation and to offer more flexibility and leeway to RET families. Irrespective of the availability of pentagastrin the calcitonin stimulation test in this setting continues to play an important role [10]. | **x** |  |  |  |  | **x** |  | **x** |  |  | **x** |  | **x** |  |  | **x** |
| **27.**  **When prophylactic thyroidectomy is delayed beyond the fifth year of life, Ct should be measured:** | **1** | **2** | **3** | **4** | **5** | **6** | **7** | **8** | **9** | **10** | **11** | **12** | **13** | **14** | **15** | **16** |
| A. every 6 months |  |  |  |  |  |  |  |  |  |  |  |  |  |  |  |  |
| B. every year | **x** |  |  |  |  |  | **x** | **x** | **x** |  | **x** |  | **x** | **x** |  | **x** |
| C. every year until age of 20, then every 2-3 years |  |  |  |  |  | **x** |  |  |  |  |  |  |  |  |  |  |
| **28.**  **Ad R6-8. Please select the level of Ct which is allowed to delay the prophylactic/**  **preemptive thyroidectomy in ATA A and B RET mutation carriers, who have less aggressive family history** | **1** | **2** | **3** | **4** | **5** | **6** | **7** | **8** | **9** | **10** | **11** | **12** | **13** | **14** | **15** | **16** |
| A. At normal basal Ct (≤10 ng/L) and normal stimulated Ct (≤ 30 ng/L) - **depending on Ct method and ranges** |  |  |  |  |  | **x** | **x** | **x** |  |  | **x** |  | **x** | **x** |  | **x** |
| B. At normal basal Ct (≤10 ng/L) and only slightly elevated stimulated Ct (≤50 ng/L) |  |  |  |  |  |  |  |  | **x** |  |  |  |  |  |  |  |
| C. At normal basal Ct (≤10 ng/L) and only moderately elevated stimulated Ct (≤ 100 ng/L) | **x** |  |  |  |  |  |  |  |  |  |  |  |  |  |  |  |
| D. I do not see the role for stimulated calcitonin estimation in this setting, normal basal Ct is sufficient |  |  |  |  |  |  |  |  |  |  |  |  |  |  |  |  |
| **29.**  **Do you agree to complete ETA comments with the statement: It is also of high importance that high volume surgeons perform pre-emptive thyroidectomies with or without additional lymph node dissection** | **1** | **2** | **3** | **4** | **5** | **6** | **7** | **8** | **9** | **10** | **11** | **12** | **13** | **14** | **15** | **16** |
| A. Yes | **x** |  |  |  |  |  |  | **x** |  |  |  |  | **x** |  |  |  |
| B. No, children should be operated by pediatric surgeons only |  |  |  |  |  |  |  |  |  |  |  |  |  |  |  |  |
| C. It depends on the experience of the given centre, however, it is important to stress prophylactic/preemptive thyroidectomy should be performed only in experienced tertiary reference centers |  |  |  |  |  | **x** | **x** |  | **x** |  | **x** |  |  | **x** |  | **x** |
| **30.**  **Guideline R10 recommends to consider RET testing in all patients with Hirschprung disease. Do you agree?** | **1** | **2** | **3** | **4** | **5** | **6** | **7** | **8** | **9** | **10** | **11** | **12** | **13** | **14** | **15** | **16** |
| A. Regular RET testing in all Hirschprung disease cases is justified to rule out cancer predisposition |  |  |  |  |  |  |  |  |  |  | **x** |  |  | **x** |  |  |
| B. Hirschsprung disease (HD) is very common (about 1/5000 births) and at least 10 related-genes have been identified. Activating RET mutations have been found in exons 10 and 11 only in in about 2% of cases (Amiel et al. 2008 [14]) and this prevalence is too low to recommend the testing as grade A recommendation | **x** |  |  |  |  | **x** | **x** | **x** |  |  |  |  |  |  |  | **x** |
| C. Further research is necessary to disclose the significance of testing for activating mutations in Hirschprung disease |  |  |  |  |  |  |  |  | **x** |  |  |  | **x** |  |  |  |
| **31.**  **Ad R11 – Considering recommendation to perform RET testing either as single or multicentre approach and its extent I prefer the statement:** | **1** | **2** | **3** | **4** | **5** | **6** | **7** | **8** | **9** | **10** | **11** | **12** | **13** | **14** | **15** | **16** |
| A. The text of ATA R11 is optimal: Analysis of the MEN 2-specific exons of *RET* is the recommended method of initial testing in either a single or multi-tiered approach. |  |  |  |  |  |  |  |  |  |  |  |  |  | **x** |  |  |
| B. Systematic screening for RET mutations in exon 10, 11, 13, 14, 15, and 16 represents the current gold standard and should be completed by exon 8 analysis in all regions where it was described to be present | **x** |  |  |  |  | **x** | **x** | **x** |  |  |  |  |  |  |  | **x** |
| C. Systematic screening for RET mutations in exon 8, 10, 11, 13, 14, 15 and 16 should be performed in all patients diagnosed with MTC |  |  |  |  |  |  |  |  | **x** |  | **x** |  |  |  |  |  |
| **32.**  **Ad R12 –If the routine analysis is negative in the clinical setting of MEN 2 or when there is a discrepancy between the genotype and phenotype, do you agree with recommendation to perform sequencing of the entire coding region of *RET* to identify MTC causative mutations:** | **1** | **2** | **3** | **4** | **5** | **6** | **7** | **8** | **9** | **10** | **11** | **12** | **13** | **14** | **15** | **16** |
| A. Yes |  |  |  |  |  |  | **x** |  | **x** |  | **x** |  | **x** | **x** |  |  |
| B. No, because this would have a poor cost effectiveness since there are 14 “remaining” exons (1, 2, 3, 4, 5, 6, 7, 9, 12, 17, 18, 19, 20, 21) and activating mutations in those exons have never been reported (see The Human Gene Mutation database, 2009 [21]) | **x** |  |  |  |  |  |  |  |  |  |  |  |  |  |  |  |
| C. There is no need to sequence the remaining exons with the exception to look for rare mutations in exon 5 (R321G) |  |  |  |  |  | **x** |  | **x** |  |  |  |  |  |  |  |  |
| **33.**  **Conflicting results have been published in recent years about the possible role of RET polymorphisms as genetic modifiers, either in sporadic or hereditary MTC.** | **1** | **2** | **3** | **4** | **5** | **6** | **7** | **8** | **9** | **10** | **11** | **12** | **13** | **14** | **15** | **16** |
| A. I propose to add in ETA comments: No definite clinical significance can be given to the presence/absence of RET polymorphic variants and no further research seems necessary | **x** |  |  |  |  |  |  |  |  |  |  |  |  | **x** |  |  |
| B. The data are still insufficient to draw any conclusions |  |  |  |  |  | **x** | **x** | **x** | **x** |  | **x** |  | **x** |  |  | **x** |
| **34. The ATA R20 recommends preoperative Ct testing in children-RET carriers with the exception of the MEN2B carriers younger than 6 month old** | **1** | **2** | **3** | **4** | **5** | **6** | **7** | **8** | **9** | **10** | **11** | **12** | **13** | **14** | **15** | **16** |
| A. It is unclear why children younger than age 6 months should not have Ct assessment. Ct assessment should be standard in all patients preoperatively. I prefer to criticize this recommendation in ETA comments |  |  |  |  |  |  | **x** | **x** |  |  | **x** |  | **x** | **x** |  | **x** |
| B. I agree with the ATA recommendation. |  |  |  |  |  | **x** |  |  |  |  |  |  |  |  |  |  |
| C. Because the reference ranges for the smallest children are not well set, it is rationale to perform the prophylactic thyroidectomy as early as possible and the Ct level does not matter. | **x** |  |  |  |  |  |  |  | **x** |  |  |  |  |  |  |  |
| **35.**  **ATA guidelines state that prophylactic level VI central compartment neck dissection may not be necessary in RET germline positive patients who undergo prophylactic thyroidectomy unless there is clinical or radiological evidence of lymph node metastases or thyroid nodules > 5 mm in size at any age, or a basal serum Ct > 40 pg/ml., because lymph node metastases are uncommon under the age of 11 years (3%), and when they are present their resection may result in long-term biochemical remission in only about one third of these patients, while 6% of all children undergoing central neck dissection may suffer hypoparathyroidism.** | **1** | **2** | **3** | **4** | **5** | **6** | **7** | **8** | **9** | **10** | **11** | **12** | **13** | **14** | **15** | **16** |
| A. I agree with the ATA guidelines in this matter |  |  |  |  |  |  |  |  |  |  | **x** |  |  | **x** |  |  |
| B. I agree with Dralle and Machens, 2009 [12], the introduction of absolute thresholds—primary tumor size of 5mm and basal calcitonin levels of 40 pg/mL—is foreign to the concept of cancer as a biologic continuum. Because they were extrapolated from a single study, these proposed cut-offs will need further evaluation before informing decisions with regard to the individualization of prophylactic thyroidectomy. Progression of hereditary medullary thyroid cancer is a stepwise process in which somatic secondary hit mutations and modifying factors mould the individual tumor phenotype from an inherited genetic background | **x** |  |  |  |  |  |  |  | **x** |  |  |  |  |  |  |  |
| C. The “5 mm” rule is too arbitrary. Even if current studies have shown a very low risk for lymph node metastases in medullary thyroid cancer (MTC) < 5 mm, it is absolutely unclear whether this fact holds true in infants with very small thyroids. Since the size of thyroid nodules and basal or stimulated calcitonin levels do not accurately predict occurrence of lymph node metastases [9] we believe that this should be stated with more caution. Especially in patients over the age of 11, prophylactic central compartment dissection still has an important role and offers the only chance of cure. |  |  |  |  |  |  | **x** | **x** |  |  |  |  | **x** |  |  |  |
| D. Central LND should be always performed with prophylactic thyroidectomy in MEN2B RET mutation carriers (ATA class D), in mutations of class A-C more flexible rules are possible |  |  |  |  |  |  | **x** |  |  |  |  |  |  |  |  |  |
| E. Central LND should be always performed with prophylactic thyroidectomy in all germline RET mutation carriers |  |  |  |  |  | **x** |  |  |  |  |  |  |  | **x** |  |  |
| **36.**  **R38 and R42 state that in asymptomatic MEN 2A and FMTC patients who present at age >5 years and asymptomatic MEN 2B patients who present at age >1 year, further evaluation prior to surgery, and more extensive surgery, is needed if the basal serum Ct is >40 pg/ml, if thyroid nodules are > 5 mm, or if suspicious lymph nodes are identified on neck US.** | **1** | **2** | **3** | **4** | **5** | **6** | **7** | **8** | **9** | **10** | **11** | **12** | **13** | **14** | **15** | **16** |
| A. I agree with the ATA guidelines |  |  |  |  |  |  |  |  |  |  |  |  |  | **x** |  |  |
| B. Preoperative basal Ct (and thyroid sonography) and thyroid sonography are always necessary and should must be completed by stimulated PG to decide whether resignation of central LND is possible (only when basal and stimulated Ct not elevated). The association of a calcitonin level < 40 ng/L with N0 status should be considered as of limited evidence. | **x** |  |  |  |  |  | **x** | **x** |  |  | **x** |  | **x** |  |  | **x** |
| C. The proposed cut-offs of basal Ct>40 ng/L and thyroid nodules of >5mm may be regarded as the cut-off limits between prophylactic (pre-emptive) surgery and therapeutic surgery but must not define the extent of surgery (the indications for central LND) |  |  |  |  |  | **x** |  |  | **x** |  |  |  |  |  |  |  |
| **37.**  **Screening for PHEO is necessary in asymptomatic RET mutation carriers** | **1** | **2** | **3** | **4** | **5** | **6** | **7** | **8** | **9** | **10** | **11** | **12** | **13** | **14** | **15** | **16** |
| A. As ATA guidelines propose, by 8^th^ year of age in ATA D class (MEN2B ) and ATA C (634 and 630) RET mutation carriers, |  |  |  |  |  |  | **x** |  | **x** |  |  |  |  | **x** |  |  |
| B. Starting at the age of 8 years seems very early as there are only a few cases of PHEO before the third decade, thus, age of 18 years seems more appropriate if there are no specific family data on earlier appearance |  |  |  |  |  |  |  | **x** |  |  | **x** |  |  |  |  |  |
| C. Always by 8th year of age in all ATA classes if an operation or pregnancy is planned | **x** |  |  |  |  | **x** | **x** |  |  |  |  |  | **x** |  |  | **x** |
| **38.**  **In MTC diagnosed/suspected preoperatively in which the familial anamnesis is negative if no RET data are available prior to surgery ATA R53 guideline recommends at least one test – biochemical or CT/MRI. Your opinion is:** | **1** | **2** | **3** | **4** | **5** | **6** | **7** | **8** | **9** | **10** | **11** | **12** | **13** | **14** | **15** | **16** |
| A. PHEO biochemical screening is mandatory in any case of diagnosed/ suspected MTC. Metanephrine screening should prioritized as the test with the highest diagnostic accuracy - comment: not verified! Depends on methods (UFR). | **x** |  |  |  |  | **x** | **x** | **x** | **x** |  | **x** |  | **x** | **x** |  | **x** |
| B. CT/MRI imaging is an alternative to exclude adrenal tumor |  |  |  |  |  |  |  |  |  |  |  |  |  |  |  |  |
| C. A+B are necessary |  |  |  |  |  |  |  |  |  |  |  |  |  |  |  |  |
| D. No additional investigations are necessary if no hypertension present |  |  |  |  |  |  |  |  |  |  |  |  |  |  |  |  |
| **39.**  **PHEO should be surgically resected after appropriate preoperative preparation and prior to surgery for MTC or PHPT. ATA R54-55 and R60 do not require scintigraphy to exclude extraadrenal pheo/malignancy. Your opinion is: *PHEO should be surgically resected after appropriate preparation and prior to surgery for MTC or PHPT. F18-DOA-PET CT is recommended to exclude bilateral/ extraadrenal/ malignant PHEO*** | **1** | **2** | **3** | **4** | **5** | **6** | **7** | **8** | **9** | **10** | **11** | **12** | **13** | **14** | **15** | **16** |
| A. I agree with ATA guideline because the risk for extraadrenal pheo is extremely small in MEN2A. Scintigraphy, either by MIBG scintigraphy or F18-DOPA is indicated only when CT/MRI is unable to localize PHEO diagnosed biochemically |  |  |  |  |  |  | **x** | **x** | **x** |  | **x** |  |  | **x** |  | **x** |
| B. I disagree, scintigraphy should be included in the preoperative assessment of PHEO in MEN2A to exclude rare cases of malignancy/multiple pheos. | **x** |  |  |  |  | **x** |  |  |  |  |  |  |  |  |  |  |
| **40.**  **At which age is screening for PHEO necessary in ATA class D (MEN2B) and ATA class C (RET 634 and 630 mutation carriers)?** | **1** | **2** | **3** | **4** | **5** | **6** | **7** | **8** | **9** | **10** | **11** | **12** | **13** | **14** | **15** | **16** |
| A. As ATA guidelines propose, by 8^th^ year of age in ATA C (634 and 630) RET mutation carriers and by 20^th^ year of age in other RET mutations associated with MEN2A |  |  |  |  |  |  | **x** |  | **x** |  | **x** |  |  | **x** |  | **x** |
| B. Starting at the age of 8 years seems very early as there are only a few cases of PHPT before the third decade, thus, age of 20 years seems more appropriate if there are no specific family data on earlier appearance, yearly checking is optimal, as the calcium estimation is a simple blood test | **x** |  |  |  |  | **x** |  | **x** |  |  |  |  |  |  |  |  |
| **41.**  **ATA R49-50 guidelines recommend surgical treatment for PHPT in MEN2A preferred to medical therapy, in the absence of contraindications such as excessive surgical risk or limited life expectancy. Your choice is:** | **1** | **2** | **3** | **4** | **5** | **6** | **7** | **8** | **9** | **10** | **11** | **12** | **13** | **14** | **15** | **16** |
| A. I follow the ATA guideline |  |  |  |  |  | **x** | **x** |  | **x** |  | **x** |  | **x** | **x** |  |  |
| B. The optimal surgical management of hyperparathyroidism in MEN2A is not yet defined. In MEN2A, especially after previous thyroidectomy, the consequences from the, frequently mild, hyperparathyroidism should be assessed per individual patient and decision for surgery reached accordingly in view of substantial rates of both permanent hypoparathyroidism as well as recurrent/ persistent hyperparathyroidism in all published series. | **x** |  |  |  |  |  |  | **x** |  |  |  |  |  |  |  | **x** |
